# Metabolomic profile of dromedary camel follicular fluid during the breeding and non-breeding seasons

**DOI:** 10.1038/s41598-025-91710-9

**Published:** 2025-03-15

**Authors:** Ahmed Sabry S. Abdoon, Seham Samir Soliman, Noha S. Hussein, Samir H. I. Haggag, Amro M. El-Sanea, Abdel-Hamid Z. Abdel-Hamid

**Affiliations:** 1 Department of Animal Reproduction and Artificial Insemination, Veterinary Research Institute, National Research Centre (NRC), Dokki, Cairo, 12622 Egypt; 2 Therapeutic Chemistry Department, National Research Centre, El-Tahrir St., Dokki, Cairo, Egypt

**Keywords:** Camel, Follicular fluid, Metabolomics, Breeding season, Non-breeding season, GC-MS, Biological techniques, Biotechnology, Genetics, Physiology

## Abstract

Understanding the metabolic profile within the follicular microenvironment is crucial for optimizing reproductive efficiency in camels. In this study, we examined the metabolomic profile of camel follicular fluid (FF) during the breeding (*n* = 10) and non-breeding seasons (*n* = 10). Gas chromatography-mass spectrometry (GC-MS) was utilized to describe the metabolites present in follicular fluid samples. The results found considerable differences in the metabolomics profiles between the breeding and non-breeding seasons. Hexadecenoic acid, galactose and glucose levels were significantly (*P* < 0.05) higher in camel FF during the breeding season, while 9-octadecenamide, oleonitrile, glycine, octadecanamide, cholesterol, and propanoic acid were higher (*P* < 0.05) in FF during the non-breeding season. Multivariante analyses pointed to those 9 metabolites, and univariate analysis showed hexadecenoic acid, galactose, glucose, and oleanitril were the most significant ones in camel follicular fluid collected during both breeding and non-breeding seasons. The univariate and multivariate analyses showed an increase in the levels of hexadecanoic acid, galactose, glucose, and a depletion in the level of oleanitrile in the breeding season compared to the non-breeding season. The ROC curve and statistical analysis showed that hexadecanoic acid, galactose, and oleanitril with AUC = 1 were promising to be seasonal biomarkers of fertility in female camels. In conclusion, the metabolomic analysis of camel FF reveals distinct changes in metabolite levels between breeding and non-breeding seasons, reflecting adaptive metabolic responses to support reproductive processes. These results offer valuable insights into the reproductive physiology of camels and offer practical implications for potential biomarkers and assessing the reproductive status in camels, which can be utilized in reproductive management and conservation efforts in these valuable animal species.

## Introduction

Camels (*Camelus dromedarius*) are unique mammals adapted to harsh desert environments; they play a vital role in the economies and cultures of many countries worldwide^1^. Camels served as a source of meat, milk, wool, and transportation, as well as a show and racing animal. Reproductive efficiency is crucial for sustaining camel populations and maximizing economic productivity. Camel reproduction is impacted by various factors, including seasonality, which is affected by the availability of resources and environmental conditions^2,3^. Seasonal variations can affect follicular development and ovulation rates, leading to distinct breeding and non-breeding seasons in camels^4,5^. Environmental factors such as diet, stress, and temperature can influence the metabolic profile of follicular fluid and impact reproductive outcomes in mammals^6–8^. Therefore, understanding the reproductive physiology of camels is vital for increasing their production and sustainability in various agricultural and pastoral systems^9^. One aspect of camel reproduction that remains relatively understudied is the metabolic changes occurring within the ovarian follicles during different reproductive seasons.

Metabolomics offers a comprehensive approach to describing the metabolite profile of follicular fluid, providing insights into the metabolic pathways and molecular mechanisms underlying ovarian follicle development and function in mammals^10^. It finds small metabolic changes that are linked to reproductive status, providing a more complete understanding of reproductive health and physiology^11^. Also, metabolomics helps find metabolic pathways and biomarkers that are unique to each species. The unique reproductive physiology and adaptability of camels to extreme environments may involve complex and multifaceted pathways that might not be possible to detect with traditional methods. Therefore, metabolomics analyses of camel follicular fluid could identify novel biomarkers and several dynamic interactions between metabolites, offering insights into metabolic shifts associated with reproduction.

The ovarian follicle is the main functioning unit of the female reproductive system, and it plays a key role in oocyte maturation and eventual fertility. Follicular fluid, the fluid surrounding the oocyte in the ovarian follicle, plays a crucial function in the maturation and development of oocytes^12^. Follicular fluid serves as a reservoir of nutrients, signaling molecules, and metabolic substrates essential for oocyte growth, development, maturation, and fertilization potentials^13^. During follicle development, alterations in metabolites are detected in energy metabolism, amino acid metabolism, lipid metabolism, and steroidogenesis, reflecting the metabolic requirements linked to the growth of follicles, proliferation of granulosa cells, and manufacture of steroid hormones^14,15^. Variations in the metabolome profiles of the follicular fluid regulate crucial processes that are essential for the follicle’s preparation and oocyte to achieve optimal fertility^16^. The stage of bovine follicular development significantly influences a total of 67 metabolites, with 33.3% reduced and 46 elevated in peri vs. preovulatory FF. The concentrations of hypoxanthine, xanthine, 17β-oestradiol, and inosine decreased. In contrast, the concentrations of retinal, 1-methyl-5-imidazoleacetate, and isovaleryl carnitine were increased^17^. Metabolomic profiling of FF could also help create biomarkers that can predict the success of bovine in vitro fertilization (IVF) and find metabolic targets that can improve the outcomes of assisted reproductive technology^18^. Researchers looked at the metabolic profile of bovine follicular fluid and found clear differences between oocytes that successfully developed into blastocysts and oocytes that broke down. They found that l-alanine, glycine, l-glutamate, and linolenic acid were higher in FF from competent oocytes, while urea, palmitic acid, and total fatty acids were much lower in FF from incompetent oocytes^19^. Furthermore, metabolomic analyses have identified metabolic signatures associated with oocyte quality, follicle maturation, and ovulation, providing valuable biomarkers for assessing reproductive health and fertility potential^20^.

Metabolomics analysis of camel follicular fluid during both the breeding and non-breeding periods might offer crucial insights into the metabolic pathways that underlie follicular growth, can help identify biomarkers associated with reproductive status and can provide valuable information for optimizing breeding strategies and enhancing reproductive efficiency. However, as far as we know, there has not been a specific study conducted on seasonal changes in the metabolomics of camel follicular fluid. Seasonal changes in camel follicular fluid could significantly influence the biochemical environment of the follicular fluid, which in turn affects follicular development, oocyte quality, and overall reproductive performance. We hypothesized that by analysing the metabolomics of camel follicular fluid, we could identify biomarkers associated with reproductive and seasonal variations. This information has the potential applications in improving reproductive management strategies, such as optimizing breeding programs, enhancing in vitro fertilization (IVF) protocols, and developing season-specific nutritional or hormonal interventions to maximize fertility. Therefore, the aim of this study was to examine the metabolomics profile of camel follicular fluid during the breeding and non-breeding seasons; we sought to identify metabolic signatures associated with reproductive seasonality in camels.

## Materials and methods

### Ethics approval and consent to participate

The in vivo experimental protocol received approval from the Institutional Animal Care and Use Committee of the National Research Centre of Egypt (Approval no: 13050416-3). The study complies with the ARRIVE guidelines and is conducted in accordance with the EU Directive 2010/63/EU for animal experiments.

### Camel follicular fluid

In this study, the ovaries were collected from twenty female camels, aged 3–7 years, who had a clinically normal reproductive tract. These camels were slaughtered over one year (October 2022 – October 2023) and were collected, representing the breeding and non-breeding seasons (10 animals per season)^21,22^. Follicular fluid (FF) is aspirated from dominant follicles of > 10 mm diameter using an 18-gauge needle attached to a 10-ml syringe. The samples were promptly centrifuged at 1000×g for 10 min to eliminate any cellular debris. The supernatant was then put into hermetically sealed glass containers and preserved at a temperature of -80 °C until it was ready for subsequent analysis.

### Sample Preparation for GC-MS analysis

The camel FF samples were thawed at room temperature for approximately 5 min and then vigorously mixed for 1 min using a vortex. 200 µl of a ternary mixture containing chloroform, methanol, and water at a ratio of 2:5:2% v/v/v was combined with 200 µl of the sample. Next, 200 µl of methanol of high-performance liquid chromatography (HPLC) quality was added, ensuring full mixing, and then vortexed for 30 s. The mixes were subjected to sonication at room temperature for approximately 30 min. The materials were thereafter transferred for centrifugation at 700×g at 4 °C. A volume of 200 µl of the sample was put into a screw-top vial with a capacity of 2 ml. The liquid thereafter underwent evaporation until it completely dried out due to the application of a nitrogen gas stream. Afterward, 100 µl of methoxyl amine hydrochloride in pyridine (20 mg/ml) was introduced, and the combination was allowed to react for 2 h at a temperature of 70 °C. Subsequently, N, O-bis (trimethylsilyl) trifluoroacetamide (BSTFA) containing 1% trimethylsilyl (TMCS) was introduced into the vial, and the resulting combination underwent a 1-hour reaction at a temperature of 40 °C^23^.

### GC-MS condition

A Thermo Gas Chromatograph coupled to an MS (Turbomass) was used to analyze camel FF. A tiny amount (1 µl) of the given treated sample was added to a PerkinElmer Clarus system that had an Elite 5MS 30.0 m × 0.25 mm internal diameter capillary column and Turbomass software. As the stationary phase, the column was 0.25 μm thick and composed of fused silica (PerkinElmer). The injector’s temperature was set to 270 °C. Helium was the carrier gas used.

### Program for capillary column temperature

The oven temperature of the GC-MS system was initially set at 40 °C. It was then increased to 200 °C at a heating rate of 5 °C and then to 300 °C at the same heating rate. Every step took about two minutes to complete. Injector and interface temperatures were controlled at 220 and 240 degrees Celsius, respectively. Helium gas, which was administered at a flow rate of 1.0 ml/min, made up the mobile phase. With a scanning range of 50–600 m/z, the electron ionization mode was used to detect MS. The multiplier’s voltage and the electrons’ energy were set to 323 V and 70 eV, respectively. The inclusion of pyridine resulted in a solvent delay of 6.5 min. Unknown chemicals were identified by comparing their spectra to those in the Wiley Library’s 2006 collection and the National Institute of Standards and Technology’s (NIST) 2005 collection. It took 58 min in total to analyze one sample^24^.

### Statistical analysis

For multivariate PLS-DA and OPLS-DA were employed to examine disparities between the seasonal and non-seasonal cohorts. To conduct univariate analysis, we perform volcano analysis (*p* < 0.01) and calculate fold change (FC). All previous analyses were performed using MetaboAnalyst 6.0 as a statistical tool. Also, the statistical analysis was conducted using the two-tailed Student’s t-test (*p* < 0.05) in SPSS 17.0 software (SPSS Inc, Chicago, IL, USA). P-value with (W) is calculated by the Wilcoxon Mann Whitney test, and the more significant the less false discover rate (FDR).The purpose was to assess the statistical significance of the results between volcano test and T- test. Finally, compounds having VIP values greater than 1.0 in OPLS-DA and univariate analysis (two-tailed student’s t-test p values less than 0.05& volcano results) were identified as prospective discriminant metabolites using a Venn diagram. The analysis was conducted utilizing the website http://www.metaboanalyst.ca. The diagnostic efficacy of putative metabolic biomarkers was evaluated using Receiver Operating Characteristic (ROC) curves, with the software MetaboAnalyst 6.0. Additionally, binary logistic regression was conducted using SPSS 17.0 software to determine the optimal combination of follicular biomarkers. (SPSS Inc., USA). In addition, a heat map and pathway analysis were conducted using MetaboAnalyst 6.0 (http://www.metaboanalyst.ca) and the Kyoto Encyclopedia of Genes and Genomes (KEGG; http://www.kegg.jp) to provide an overview of the metabolites that showed significant changes. Pathways that have raw p values less than 0.05 and impact values greater than 0 were considered as possible biomarkers.

### Follicular metabolite profiling by GC-MS

The workflow of metabolomics research of follicular fluid for breeding and non-breeding seasons, validating biomarker discovery and establishment. The metabolomics species obtained from follicular fluid were effectively isolated and examined using GC-MS. Finally, these metabolites varied from sugars, amino acids, and lipid metabolic pathways.

### Multivariate statistical analysis

**Fig. 1:**
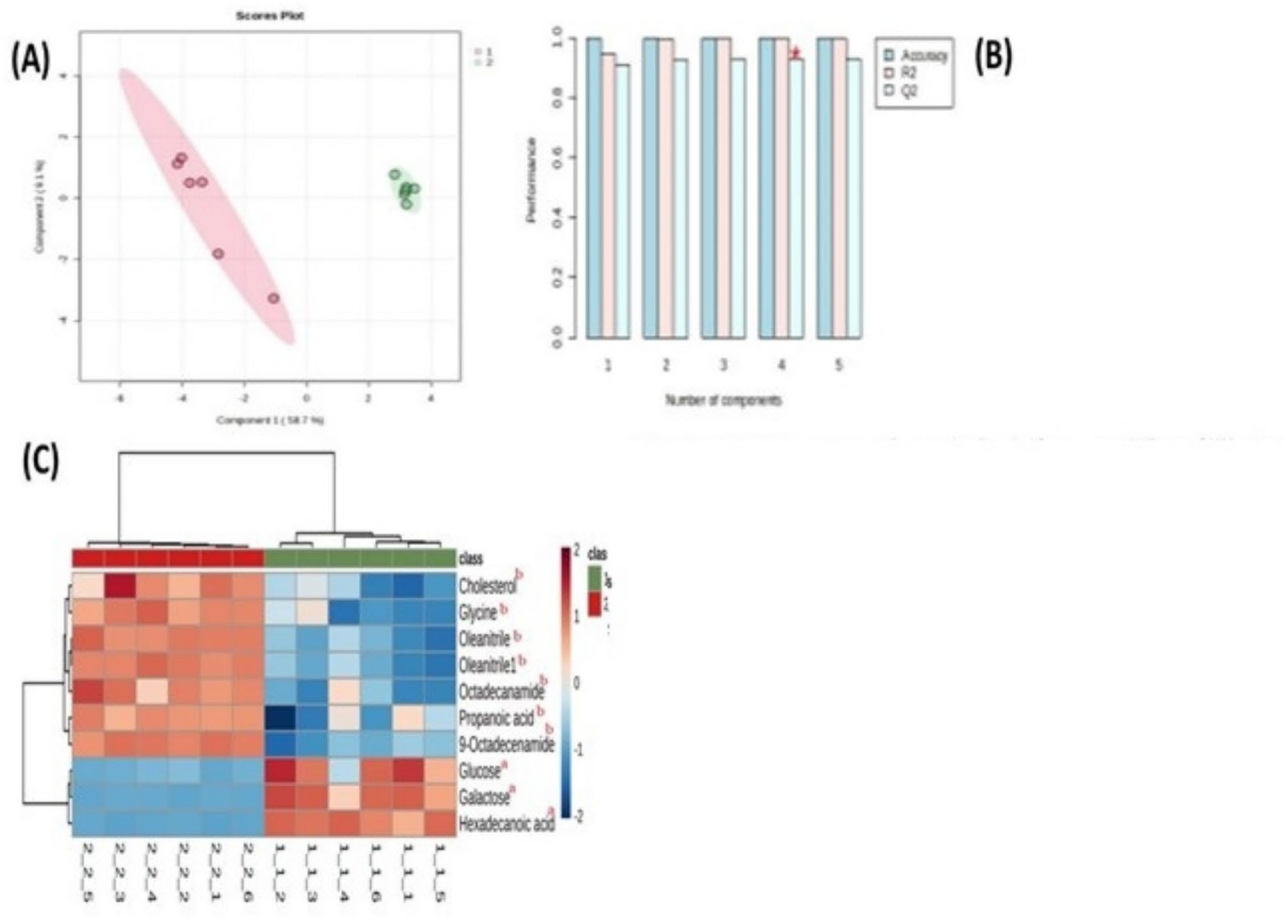
PLS-DA score plots obtained from modeling follicular metabolites in the breeding & non-breeding seasons. (**A**) The score plot showing a separation of FF collected during breeding (1) & non-breeding seasons (2) in camels; (**B**) Hierarchical clustering a0nalysis of metabolites in FF collected during breeding & non-breeding seasons, (**C**) The permutation test of PLS-DA model, respectively. The heat map of metabolomics metabolites indicates the increase or decrease of each metabolite in each group with classes as 1 Breeding & non-breeding season 2. The top 9 metabolites according to VIP of PLS-DA in breeding & non-breeding season were determined from down to top. ^a^Indicated to metabolite clusters increased in seasonal group. ^b^indicated to metabolite clusters decreased in seasonal group. Data transformation is done using log transformation especially Pareto-scaling option prior to multivariate data analysis.

A Partial Least Squares Discriminant Analysis (PLS-DA) model was created using metabolomics profiling data obtained from both breeding season and non-breeding season modes. The PLS-DA model yielded the first two principal components, PC1 and PC2. Figure 1A demonstrates that the follicular fluids of seasonal camels distinctly differ from those of non-seasonal camels in the direction of the PC1. The R2Y and Q2 (cumulative) model values were 98% and 97%, respectively, demonstrating satisfactory fitting and predictive capability for the PLS-DA model (Fig. 1B). In addition, response permutation testing was conducted with a sample size of 100, which showed that the model did not suffer from overfitting, as shown by a P-value of less than 0.05. Hence, the multivariate analysis demonstrated that the PLS-DA model exhibited a considerable level of reliability and potential for identifying reproductive biomarkers in follicular secretions.

## Results

A preliminary analysis of the metabolomics data revealed significant differences in the metabolomics profiles of camel follicular fluid between the breeding and non-breeding seasons. Several metabolites exhibited seasonal variations, with some being more abundant during the breeding season, while others were elevated during the non-breeding season. Key metabolites associated with reproductive processes, such as amino acids and lipids, showed distinct patterns of abundance between the two seasons.

### Multivariate statistical analysis

**Fig. 2:**
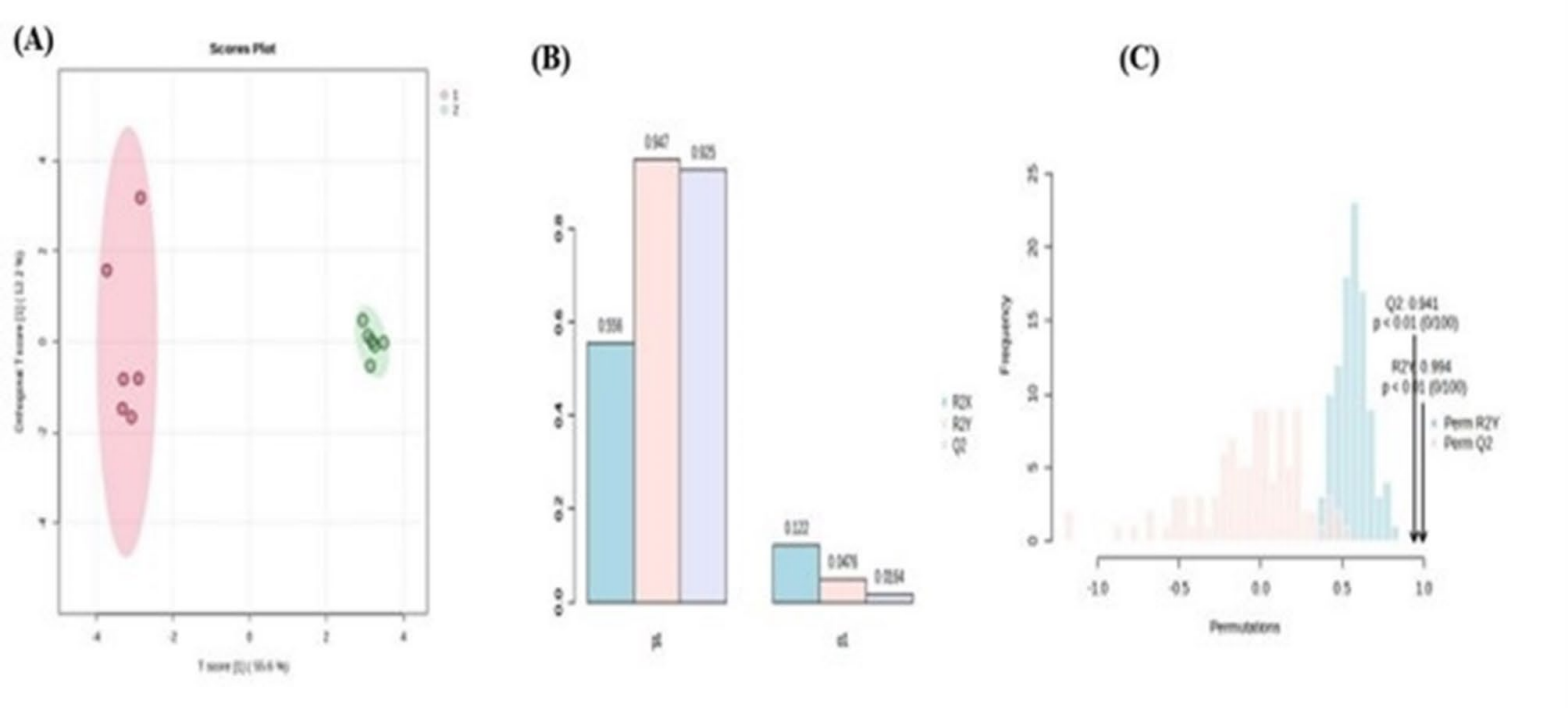
OPLS-DA score plots obtained from modelling follicular metabolites in the breeding and non-breeding seasons. (**A**): The score plot showing a separation of breeding (1) & non-breeding (2) seasons; (**B**, **C**): The permutation test of the OPLS-DA model. Data transformation is done using log transformation especially Pareto-scaling option prior to multivariate data analysis.

A Partial Least Squares Discriminant Analysis (PLS-DA) model was developed using metabolomics profiling data obtained from follicular fluid samples collected during both breeding and non-breeding seasons. The PLS-DA model yielded the first two principal components, PC1 and PC2. As depicted in Fig. 2A, follicular fluids of camels during the breeding season displayed a clear separation from the non-breeding season in the direction of PC1. The R2 and Q2 (cumulative) model values were 98% and 97%, respectively, demonstrating a satisfactory level of fitting and predictive capability for the PLS-DA model (Fig. 2c). In addition, response permutation testing was conducted with a sample size of 100, which showed that the model did not suffer from overfitting, as shown by a P-value of less than 0.05. Hence, the multivariate analysis demonstrated that the PLS-DA model had a considerable degree of reliability and potential in identifying reproductive biomarkers in follicular secretions. In the multivariate analysis PLS-DA, only species with variable importance (VIP) values higher than 1 were kept, as they made significant contributions to the categorization. According to the PLS-DA model, nine metabolites with VIP > 1 were selected as candidate biomarkers. These metabolites are hexadecanoic acid, galactose, 9-octadecenamide, oleonitril, glycine, glucose, cholesterol, octadecanamide, and propanoic acid. An overview of nine metabolites that experienced substantial changes was generated using a heat map analysis of the Pearson correlation coefficients (Fig. 2B).

The heat map of metabolomics metabolites indicates the increase or decrease of each metabolite in each group with classes as1 Breeding and non-breeding season 2. The top 9 metabolites according to VIP of PLS-DA in breeding and non-breeding season were determined from down to top. S-DA analyses, there were clear distinctions between the FF collected during the breeding & non-breeding season. The model parameters achieved scores that were approximately 1.0. (R^2^ X = 0.556, R^2^ Y = 0.947, Q^2^ = 0.925), signifying that the model was effective and distinctly distinguished the FF of breeding and the non-breeding season. The permutations plot confirmed the validity of the original model because the Q2 value = 0.941, near to one with *P* < 0.01, also, the R^2^ Y = 0.994, near to one with *P* < 0.01 (Fig. 1A–C).

The outcomes of the cluster analysis on the expression of metabolites in follicular fluids in the breeding season and non-breeding season are shown in Fig. 1. The sample clusters exhibited a small overlap during the non-breeding season group, the metabolite content concentration in the breeding season group was notably different from that in the non-breeding season group, signifying that the samples exhibited excellent consistency, and the data was trustworthy.

**Fig. 3:**
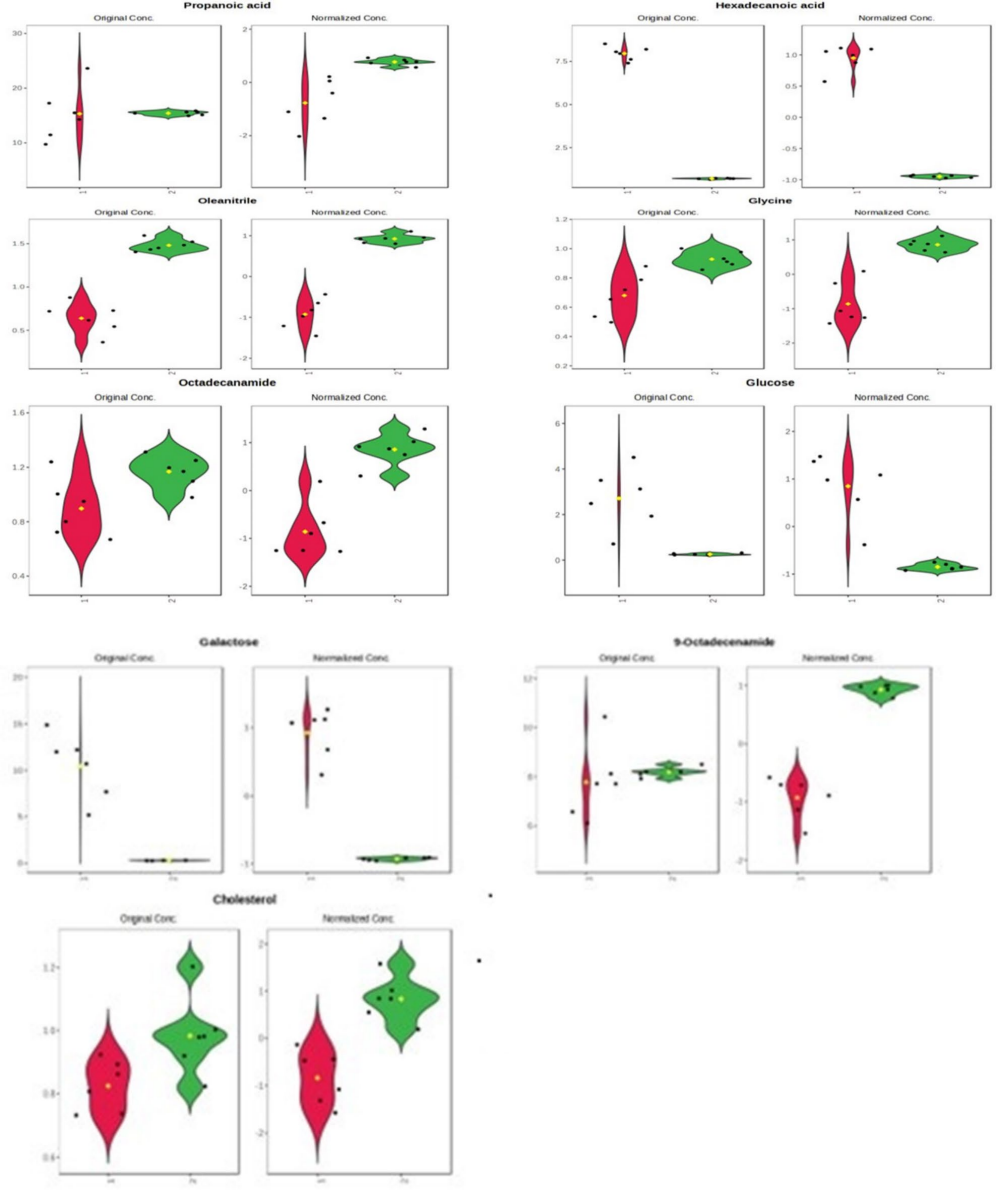
Violin plot of metabolites derived from VIP of the OPLS-DA model. 1: indicating FF of the breeding season (red colour) and 2: indicating FF of the non-breeding season (green colour).

For OPLS-DA metabolites of VIP > 1, they are the same as mentioned in PLS-DA, noticing that the increased levels of hexadecanoic acid, glucose, and galactose in FF collected during the breeding season and the increase in 9-octadecenamide, oleonitril, glycine, octadecanamide, cholesterol, and propanoic acid in the non-breeding season group when compared with the breeding one as mentioned in the violin plot Fig. 3. Finally, 9 metabolites were selected by multivariate analysis, including PLS-DA and OPLS-DA.

**Fig. 4:**
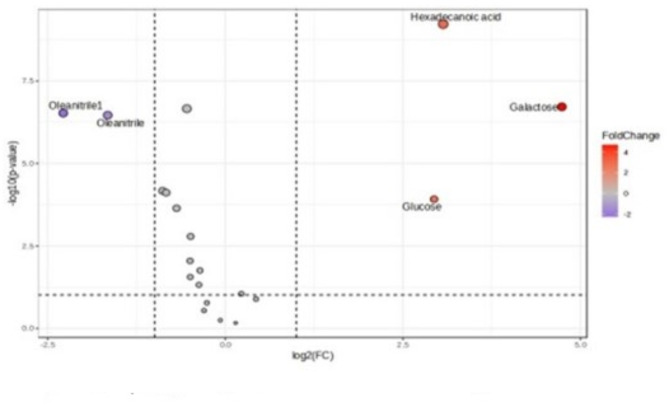
Volcano plot of follicular fluid metabolomics data.

For univariate analysis, volcano analysis was performed, indicating 4 statistically significant metabolites (*p* < 0.01) were selected for further validation when comparing follicular fluid collected during the breeding season with the non-breeding seasonal group in female camels as shown in Fig. 4. The plot shows an increase in the levels of hexadecanoic acid, galactose, and glucose and an increase in the level of oleanitril in the FF of the non-breeding season group compared to the breeding seasonal group.

In addition, the student’s t-test two-tailed analysis using SPSS Ver. 17 indicated that 10 metabolites significantly differ between FF of breeding and non-breeding season groups with *P* values less than 0.05. These metabolites include aceton, glycine, proline, hexadecanoic acid, galactose, glucose, hexadecanamide, octadecinamide, oleonitrile, and cholesterol.

**Fig. 5:**
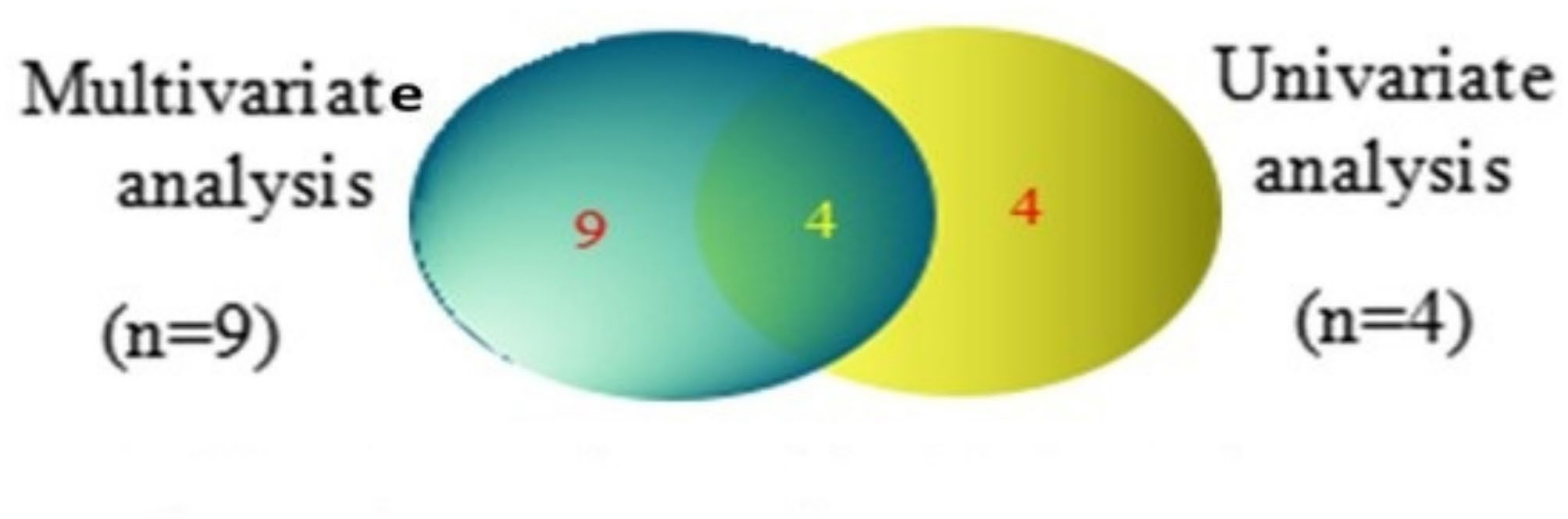
Pathway analysis diagram performed using MetaboAnalyst 6.0. The red color indicates the more significant pathways at *p* < 0.01.

Finally, four metabolites were selected by univariate analysis, including volcano and Student’s t-test, including hexadecanoic acid, glucose, galactose, and oleanitrile. A Venn plot of multivariate analysis and univariate analysis was performed and showed that hexadecanoic acid, glucose, galactose, and oleanitrile metabolites are promising biomarkers, as shown in Fig. 5.

### Validation of metabolomic biomarkers

To verify the precision of these three potential metabolic fertility indicators in distinguishing the metabolomics of FF obtained during the reproductive and non-reproductive periods. These three possible biomarkers are needed to fulfill the following criteria. First, the VIP of PLS-DA and VIP of OPLS-DA values of these four metabolite species must be simultaneously greater than 1. Hexadecanoic acid, glucose, galactose, 9-octadecenamide, oleonitril, glycine, octadecanamide, cholesterol, and propanoic acid were met and set aside for additional verification. Secondly, potential species must exhibit simultaneous statistical dissimilarity (*p* < 0.05) between FF collected during the breeding and non-breeding season. Glycine, hexadecanoic acid, galactose, glucose, oleonitrile, and cholesterol were verified successfully (Table 1). Finally, FC values of candidate metabolite species must be simultaneously more than 1.5 or less than 0.5 in both comparison groups. Four metabolites, including hexadecanoic acid, glucose, galactose, and oleanitrile met this standard (Table 1).

**Table 1:** Statistical information of candidate metabolite biomarkers.

			Hexadecanoic acid	Galactose	Oleonitrile	Glucose
Validation set						
Multivariate	S vs. NS	VIP[1]P	1.33	1.305	1.301	1.19
		VIP[2]O	1.32	1.306	1.305	1.20
Univariate	S vs. NS	*P*	5.99 × 10^− 10^	1.93 × 10^− 7^	2.98 × 10^− 7^	1 × 10^− 4^
		*Adjusted P*	9.22	6.71	6.52	3.91
		FC	8.37	26.66	0.205	7.66
		Trend	*P* < 0.001↑	*P* < 0.001↑	*P* < 0.001↓	*P* < 0.001↑

The upward arrow (green color) signifies that the level of metabolites in the seasonal group was increased compared to the non-seasonal group. The downward arrow (red color) signifies that the concentration of metabolites in the non-breeding season group was higher compared to the breeding season group.

### Assessment of the diagnostic accuracy of potential metabolic biomarkers

Following the confirmation of prospective biomarkers in the validation set, an ROC curve was employed to evaluate the diagnostic capacity of these four metabolic biomarkers. According to the information presented in Table 2, only the AUC value of hexadecanoic acid, galactose, glucose, and oleanitril reached 1.0 when comparing the FF collected during the breeding and non-breeding season. However, the ability of glucose metabolite to differentiate the breeding from non-breeding seasons, to which we want to pay greater attention, was unsatisfactory because of a partial lower *P* value compared to other compound P values.

**Table 2:** The results of ROC analysis for fertility metabolic biomarkers.

Metabolites	AUC	SE	95% cl		*P* value	Sensitivity	Specificity
			Lower	Upper			
Hexadecanoic Acid	1.0	0.285	1.00	1.00	0.6 × 10^− 11^	1.0	1.0
Galactose	1.0	2.455	1.00	1.00	0.1 × 10^− 10^	1.0	1.0
Oleanitril	1.0	0.139	1.00	1.00	0.2 × 10^− 10^	1.0	1.0
Glucose	1.0	0.577	1.00	1.00	0.1 × 10^− 9^	1.0	1.0

Consequently, a binary logistic regression using forward stepwise selection (Wald) was conducted to determine the optimal combination of these metabolic biomarkers. The findings revealed that a panel consisting of hexdecanoic acid, galactose, and oleanitril was the most effective combination of these three metabolic species (Table 3), and without galactose, the *P* value was increased from 0.008 to 0.031 indicating that the combination set of three metabolites was stronger and better than the individual metabolite for differentiating between FF collected during the breeding and non-breeding seasons in camels.

**Table 3:** Binary logistic regression results of the biomarkers set.

			Score	df	Sig				B	SE	Wals	df	Sig
Variables not in the model						Variables in the model							
Step 0	Variables	Hexadecanoic acid	4.940	1	0.026	Step 0	Variables	Not allowed					
	Overall statistics	Galactose	5.196	1	0.023								
		Oleanitril	2.884	1	0.089								
			11.914	3	0.008								
Step 1	Variables	Hexadecanoic acid	0.886	1	0.346	Step1	Galactose		-0.45	0.39	1.314	1	0.252
	Overall-statistics	Oleanitril	6.847	1	0.009								
			6.934	2	0.031								

### Analyses of metabolic pathways

**Fig. 6:**
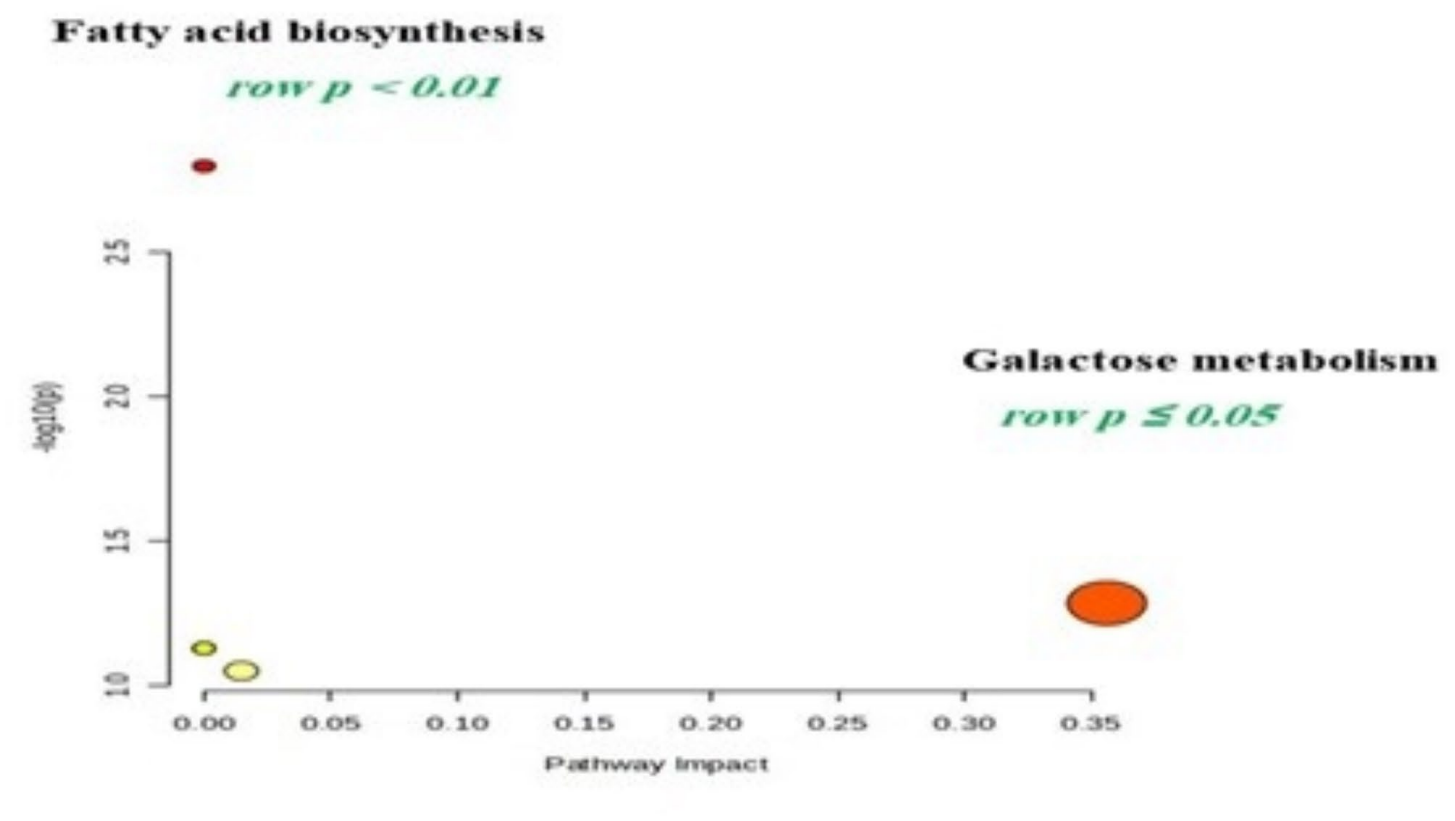
Pathway analysis diagram performed using MetaboAnalyst 6.0. The red color indicates the more significant pathways.

Several metabolic pathways exhibited a strong correlation with fertility during the breeding season in female camels. The pathways of strong significance identified in the follicular fluid were fatty acid biosynthesis, saturated (palmitic acid) & unsaturated (oleic acid) and galactose metabolism, respectively, as shown in Fig. 6. Additional details are shown in Table 4.

**Table 4:** Metabolic pathways related to female camel fertility intervention mechanisms in the seasonal group.

Pathway name	Match status	Raw *P*	FDR	Impact
Biosynthesis of unsaturated fatty acids	2/36	0.0015898	1.27E-01	0.00
Galactose metabolism	1/27	0.052012	1.00E + 00	0.35614
Fatty acid elongation	1/39	0.074538	1.00E + 00	0.00
Fatty acid degradation	1/39	0.074538	1.00E + 00	0.00
Fatty acid biosynthesis	1/47	0.089355	1.00E + 00	0.01473

*Raw p* the original p value calculated from the enrichment analysis, *FDR* false discovery rate. The impact is the pathway impact value calculated from pathway topology analysis.

## Discussion

Metabolomics is a relatively new field, and while there is a growing interest in applying it to various biological systems, including reproductive biology, there may not yet be comprehensive data available on the metabolomics of camel follicular fluid specifically. Monitoring these metabolic changes could aid in predicting optimal breeding periods, assessing reproductive performance, and implementing targeted interventions to enhance fertility and reproductive efficiency in camel herds.

In the present work, the observed changes in metabolite abundance and metabolic pathway activation reflect the dynamic nature of ovarian physiology in dromedary camels and its regulation by seasonal cues, which could be due to metabolic adaptations occurring during different reproductive seasons. During the breeding season, which extends from October to the end of April, increased availability of nutrients and energy substrates may promote enhanced follicular development and oocyte maturation. Conversely, during the non-breeding season, metabolic pathways are redirected towards maintenance and survival rather than reproduction, leading to alterations in follicular fluid composition. The present results showed that the levels of hexadecanoic acid (palmitic acid), glucose, and galactose significantly (*P* < 0.05) increased in camel FF during the breeding season, while the levels of 9-octadecenamide, oleonitrile, glycine, octadecanamide, cholesterol, and propanoic acid were significantly (*P* < 0.05) higher in FF collected during the non-breeding season compared to the breeding season. The elevated levels of hexadecanoic acid, glucose, and galactose in FF collected during the breeding season may indicate a metabolic shift towards increased energy production and utilization. They achieve this by increasing the energy supply and providing the necessary building blocks to produce important hormones, such as steroid hormones and prostaglandins, which play a crucial role in reproductive processes. Glucose is crucial for ovarian metabolism as it serves as the primary energy source for ovary. Through glycolysis and oxidative phosphorylation, glucose provides ATP essential for cellular processes, including meiosis and maturation^25^. Cumulus cells metabolize glucose to pyruvate and lactate, which are directly utilized by oocytes^26^. Also, the small ovarian follicles possess the capacity to selectively extract and store the elevated levels of glucose from the bloodstream for their growth into the fully developed Graafian follicle. Glucose and metabolic hormones have a direct impact on the regulation of steroidogenesis at the ovarian level^27^. A reduction in the amount of glucose present in the pre-ovulatory follicle fluid that was growing in cows and mares from a dominant follicle^28^. The larger follicles in dairy cows were associated with higher glucose levels in the follicular fluid^29^. In addition, our findings demonstrate a noteworthy elevation in galactose levels throughout breeding seasons, suggesting the presence of other carbohydrates in follicular fluids apart from glucose. Galactose contributes to glycoprotein and glycolipid biosynthesis, which are integral for cell signalling and membrane integrity in follicular cells and oocyte^30^. The utilization of galactose for glycolysis within the follicle might lead to a substantial accumulation of this sugar. Its presence in follicular fluids may influence carbohydrate metabolism^31^. The interaction of glucose and galactose in follicular fluid is crucial for ensuring the developmental competence of oocytes, influencing their ability to progress through fertilization and embryonic development, and this is essential for creating an environment that supports oocyte metabolism, growth, and developmental potential^32^. Maintaining a delicate balance of these monosaccharides is critical for ensuring optimal oocyte viability and successful reproductive outcomes^33^. In contrast, mouse oocytes exposed during in vitro maturation to 2µM D-galactose, and its metabolites disturbed the spindle structure and chromosomal alignment, which was associated with significant decline in oocyte cleavage and blastocyst development after in-vitro fertilization^34^. This discrepancy could be due to species differences.

In addition, functional fatty acids and their byproducts enhance the growth of follicles, maturation of eggs, development of embryos, and the receptivity of the uterus lining^35^. Hexadecanoic acid (commonly known as palmitic acid) is a saturated fatty acid that plays a crucial role in metabolic processes. Its role in energy production is primarily mediated through beta-oxidation, a process that occurs in the mitochondria. In steroidogenesis, palmitic acid serves as a precursor for cholesterol biosynthesis. Through the activity of the enzyme acetyl-CoA carboxylase, acetyl-CoA derived from palmitic acid is converted into malonyl-CoA, a key building block for fatty acid synthesis. Further elongation and desaturation processes contribute to the synthesis of cholesterol, a critical substrate for steroid hormone production. These pathways highlight the multifaceted roles of palmitic acid in both cellular energy homeostasis and the biosynthesis of biologically active lipids^36,37^. Hexadecenoic acid is known to influence reproductive physiology by modulating prostaglandin synthesis, improving oocyte quality, and enhancing the uterine environment. It has been incorporated into diets to boost reproductive performance. On the other hand, a study on oocytes demonstrated that elevated concentrations of non-esterified fatty acids, including palmitic acid, during in vitro maturation of human oocytes impaired developmental competence, leading to blastocysts with lower cell numbers and increased apoptosis^38^. This discrepancy may be related to species difference or the culture conditions.

In dromedary camels, the non-breeding season is associated with significant changes in their reproductive physiology, often regulated by hormonal fluctuations and metabolic adjustments. The increased levels of certain metabolites, such as 9-octadecenamide, oleonitrile, glycine, octadecanamide, cholesterol, and propanoic acid, in follicular fluid (FF) during non-breeding season might play distinct roles in reproductive function. These metabolites play diverse roles in lipid metabolism, signaling cascades, and cellular homeostasis. During the non-breeding season, elevated octadecanamide levels might influence ovarian quiescence by reducing follicular activity. Its involvement in stress and energy homeostasis could also help camels adapt to challenging environmental conditions. While the increase in oleonitrile may indicate shifts in lipid metabolism during the non-breeding season. This could support the maintenance of follicular integrity and regulate the balance between growth and atresia of follicles. Elevated glycine levels in FF may serve to protect ovarian follicles from oxidative stress during metabolic downregulation in the non-breeding season, and it might also support follicular cell survival by promoting glutathione synthesis and osmoregulation^39^. While the elevated cholesterol levels may reflect a reserve for potential steroidogenesis during the transition to the breeding season^40^. In sheep, during pregnancy, fetal growth depends not on the type of FA during the finishing phase but the interaction of different sources of FA and different stages. Also, supplementation with FA during early pregnancy changes productive performance and neuropeptides’ mRNA expression of lambs independently of the finishing diet^41^. Although, the lack of active ovulation in camel during the non-breeding season might lead to cholesterol accumulation within FF. Meanwhile, the increase in propanoic acid might indicate shifts in metabolic activity within the follicular microenvironment. It may serve as an alternative energy source for follicular cells and contribute to maintaining low-grade inflammation or immune tolerance^42^. These metabolic adjustments might suggest their role in maintaining follicular dormancy and preventing premature follicular development during the non-breeding season and reflect the camel’s ability to cope with seasonal variations in temperature and resource availability. The changes in metabolite levels may be linked to reduced gonadotropin activity and shifts in local ovarian signalling pathways. The observed changes in metabolite profiles between breeding and non-breeding seasons highlight the intricate interplay between metabolic pathways and reproductive physiology in camels. These seasonal metabolic adaptations likely optimize resource allocation, energy utilization, and nutrient availability to meet the specific demands of reproduction. Such metabolic reprogramming ensures optimal follicular development, ovulation, and successful reproductive outcomes during the breeding season while conserving energy and metabolic resources during periods of reproductive quiescence and preserve reproductive function for the upcoming breeding season.

## Conclusion

Metabolomic analysis of camel FF reveals distinct alterations in metabolite levels between breeding and non-breeding seasons, reflecting adaptive metabolic responses to support reproductive processes. These findings deepen our understanding of seasonal reproductive physiology in camels and offer practical implications for reproductive management and conservation efforts in these valuable animal species. Further studies are needed to elucidate the precise molecular mechanisms behind these changes.

## Data Availability

The data that support the findings of this study are available on request from the first author [ASSA].

## References

[1] Abdoon, A. S. S., Soliman, S. S. & Nagy, A. M. Uterotubal junction of the bovine (Bos taurus) versus the dromedary camel (*Camelus dromedarius*): Histology and histomorphometry. *Reprod. Domest. Anim.* **59**(7), 14665. 10.1111/rda.14665 (2024). 10.1111/rda.14665 38973694

[2] Marai, I. F. M. et al. Camels reproductive and physiological performance traits as affected by environmental conditions. *Trop. Subtrop. Agroecosyst.* **10**, 129–149 (2009).

[3] Soliman, S., Elsanea, A., Kandil, O., Aboelmaaty, A. & Abdoon, A. impact of reproductive status, body condition score, and locality on hormonal, and some blood metabolites in Egyptian buffaloes. *Egypt. J. Vet. Sci.* **55**(5), 1387–1396. 10.21608/ejvs.2024.252235.1699 (2024).

[4] Abdoon, A. S. Factors affecting follicular population, oocyte yield and quality in camels (*Camelus dromedarius*) ovary with special reference to maturation time in vitro. *Anim. Reprod. Sci.* **66**(1–2), 71–79. 10.1016/S0378-4320(01)00078-1 (2001). 11343843 10.1016/s0378-4320(01)00078-1

[5] Sghiri, A. & Driancourt, M. A. Seasonal effects on fertility and ovarian follicular growth and maturation in camels (*Camelus dromedarius*). *Anim. Reprod. Sci.* **55**(3–4), 223–237. 10.1016/S0378-4320(99)00017-2 (1999). 10379674 10.1016/s0378-4320(99)00017-2

[6] Abdoon, A. S. S. et al. Effect of reproductive status and season on blood biochemical, hormonal and antioxidant changes in Egyptian buffaloes. *Int. J. Vet. Sci.* **9**(1), 131–135. 10.5555/20203194109 (2020).

[7] Soliman, S. S. et al. Seasonal variation in ovarian functions in Egyptian buffalo and cattle. *Int. J. Pharm. Tech. Res.* **9**(6), 34–42 (2016).

[8] Abdoon, A. S. et al. Seasonal variation in number of ovarian follicles and hormonal levels in Egyptian buffalo and cattle. *Int. J. Vet. Sci.* **9**(1), 126–130 (2020).

[9] El-Sanea, A. M. & Soliman, S. S. Ovarian morphometry and follicular population in non-pregnant and pregnant camelus dromedaries. *Egypt. J. Vet. Sci.* 1–7. 10.21608/EJVS.2025.342608.2545 (2025).

[10] Ravisankar, S. et al. Metabolomics analysis of follicular fluid coupled with oocyte aspiration reveals importance of glucocorticoids in primate periovulatory follicle competency. *Sci. Rep.* **11**, 6506. 10.1038/s41598-021-85704-6 (2021). 33753762 10.1038/s41598-021-85704-6 PMC7985310

[11] Hennet, M. L. & Combelles, C. M. H. The antral follicle: A microenvironment for oocyte differentiation. *Int. J. Dev. Biol.* **56**, 819–831 (2012). 23417404 10.1387/ijdb.120133cc

[12] Revelli, A. et al. Follicular fluid content and oocyte quality: From single biochemical markers to metabolomics. *Reprod. Biol. Endocrinol.* **7**, 40. 10.1186/1477-7827-7-40 (2009). 19413899 10.1186/1477-7827-7-40 PMC2685803

[13] Izquierdo, D. et al. Fatty acids and metabolomic composition of follicular fluid collected from environments associated with good and poor oocyte competence in goats. *Int. J. Mol. Sci.* **23**, 4141 (2022). 35456957 10.3390/ijms23084141 PMC9028732

[14] Da Broi, M. G. et al. Influence of follicular fluid and cumulus cells on oocyte quality: Clinical implications. *J. Assist. Reprod. Genet.* **35**, 735–751. 10.1007/s10815-018-1143-3 (2018). 29497954 10.1007/s10815-018-1143-3 PMC5984887

[15] Zhang, C. H., Liu, X. Y. & Wang, J. Essential role of granulosa cell Glucose and lipid metabolism on oocytes and the potential metabolic imbalance in polycystic ovary syndrome. *Int. J. Mol. Sci.* **24**, 16247. 10.3390/ijms242216247 (2023). 38003436 10.3390/ijms242216247 PMC10671516

[16] Hessock, E. A. et al. Metabolite abundance in bovine preovulatory follicular fluid is influenced by follicle developmental progression post estrous onset in cattle. *Front. Cell Dev. Biol.* **11**, 1156060. 10.3389/fcell.2023.1156060 (2023). 37215073 10.3389/fcell.2023.1156060 PMC10196500

[17] Alrabiah, N. A. et al. Biochemical alterations in the follicular fluid of bovine peri-ovulatory follicles and their association with final oocyte maturation. *J. Reprod. Fertile.* **4**, 220090. 10.1530/RAF-22-0090 (2023). 10.1530/RAF-22-0090 PMC9874974 36547396

[18] McRae, C., Sharma, V. & Fisher, J. Metabolite profiling in the pursuit of biomarkers for IVF outcome: The case for metabolomics studies. *Int. J. Reprod. Med.* **16**, 603167. 10.1155/2013/603167 (2013). 10.1155/2013/603167 PMC4334075 25763388

[19] Matoba, S. et al. Predictive value of bovine follicular components as markers of oocyte developmental potential. *Reprod. Fertil. Dev.* **26**(2), 337–345. 10.1071/RD13007 (2013). 10.1071/RD13007 23514964

[20] Yang, J. et al. Metabolic signatures in human follicular fluid identify lysophosphatidylcholine as a predictor of follicular development. *Commun. Biol.* **5**, 763. 10.1038/s42003-022-03710-4 (2022). 35906399 10.1038/s42003-022-03710-4 PMC9334733

[21] Bernard, W. M. & Swanson, D. L. Metabolic profiling and integration of metabolomic and transcriptomic data from pectoralis muscle reveal winter-adaptive metabolic responses of black-capped chickadee and American goldfinch. *Front. Ecol. Evolut.* **10**, 866130. 10.3389/fevo.2022.866130 (2022).

[22] Walker, H. K. et al. Seasonal variation in serum metabolites of northern European dogs. *J. Vet. Int. Med.* **36**(1), 190–195 (2022). 10.1111/jvim.16298 PMC8783344 34921444

[23] Olivier, I. & Loots, D. T. A. metabolomics approach to characterise and identify various Mycobacterium species. *J. Microbiol. Methods* **88**(3), 419–426 (2012). 22301369 10.1016/j.mimet.2012.01.012

[24] Ahamad, S. R. et al. Metabolomics and trace element analysis camel tears by GC-MS and ICP-MS. *Biol. Trace Elem. Res.* **177**, 251–257 (2017). 27837381 10.1007/s12011-016-0889-7

[25] Xiong, Y. Y. et al. Regulation of glucose metabolism: Effects on oocyte, preimplantation embryo, assisted reproductive technology and embryonic stem cell. *Helyino* **10**(19), e38551 (2024). 10.1016/j.heliyon.2024.e38551 PMC11471579 39403464

[26] Xie, H. L. et al. Effects of glucose metabolism during in vitro maturation on cytoplasmic maturation of mouse oocytes. *Sci. Rep.* **6**, 20764. 10.1038/srep20764 (2016). 26857840 10.1038/srep20764 PMC4746733

[27] Munõz-Gutiérrez, M. et al. Ovarian follicular expression of mRNA encoding the type 1 insulin like growth factor receptor (IGF-IR) and insulin like growth factor binding protein 2 (IGFBP2) in anoestrous sheep after 5 days of glucose, glucosamine or supplementary feeding with lupin grain. *Reproduction* **128**, 747–756. 10.1530/rep.1.00439 (2004). 15579592 10.1530/rep.1.00439

[28] Iwata, H. et al. Effects of follicle size and electrolytes and glucose in maturation medium on nuclear maturation and developmental competence of bovine oocytes. *Reproduction* **127**, 159–164. 10.1530/rep.1.00084 (2004). 15056781 10.1530/rep.1.00084

[29] Leroy, J. L. et al. Metabolite and ionic composition of follicular fluid from different sized follicles and their relationship to serum concentrations in dairy cows. *Anim. Reprod. Sci.* **80**, 201–211. 10.1016/S0378-4320(03)00173-8 (2004). 15036497 10.1016/S0378-4320(03)00173-8

[30] Shivatare, S. S., Shivatare, V. S. & Wong, C. H. Glycoconjugates: Synthesis, functional studies, and therapeutic developments. *Chem. Rev.* **122**(20), 15603–15671. 10.1021/acs.chemrev.1c01032 (2022). 36174107 10.1021/acs.chemrev.1c01032 PMC9674437

[31] Pan, Y. et al. Unraveling the complexity of follicular fluid: insights into its composition, function, and clinical implications. *J. Ovarian. Res.* **17**, 237. 10.1186/s13048-024-01551-9 (2024). 39593094 10.1186/s13048-024-01551-9 PMC11590415

[32] Fontana, J. et al. Metabolic cooperation in the ovarian follicle. *Physiol. Res.* **69**(1), 33–48. 10.33549/physiolres.934233 (2019). 31854191 10.33549/physiolres.934233 PMC8565957

[33] Gu, L. et al. Metabolic control of oocyte development: Linking maternal nutrition and reproductive outcomes. *Cell. Mol. Life Sci.* **72**, 251–271 (2015). 25280482 10.1007/s00018-014-1739-4 PMC4389777

[34] Thakur, M. et al. Galactose and its metabolites deteriorate metaphase II mouse oocyte quality and subsequent embryo development by disrupting the spindle structure. *Sci. Rep.* **7**(1), 231. 10.1038/s41598-017-00159-y (2017). 28331195 10.1038/s41598-017-00159-y PMC5427935

[35] Zeng, X. Z. et al. Role of functional fatty acids in modulation of reproductive potential in livestock. *J. Anim. Sci. Biotechnol.* **14**, 24. 10.1186/s40104-022-00818-9 (2023). 36788613 10.1186/s40104-022-00818-9 PMC9926833

[36] Prakash, S. Beta (β)-Oxidation of fatty acid and its associated disorders. *Int. J. Clin. Biochem.* **5**(1), 158–172 (2018).

[37] Hu, J. et al. Cellular cholesterol delivery, intracellular processing and utilization for biosynthesis of steroid hormones. *Nutr. Metab. (Lond.)* **7**, 47. 10.1186/1743-7075-7-47 (2010). 20515451 10.1186/1743-7075-7-47 PMC2890697

[38] Uhde, K. et al. Metabolomic profiles of bovine cumulus cells and cumulus-oocyte-complex-conditioned medium during maturation in vitro. *Sci. Rep.* **8**, 9477. 10.1038/s41598-018-27829-9 (2018). 29930262 10.1038/s41598-018-27829-9 PMC6013446

[39] Gao, L. et al. Glycine regulates lipid peroxidation promoting porcine oocyte maturation and early embryonic development. *J. Anim. Sci.* **101**, 425. 10.1093/jas/skac425 (2023). 10.1093/jas/skac425 PMC9904182 36573588

[40] Jungheim, E. S. et al. Associations between free fatty acids, cumulus oocyte complex morphology and ovarian function during in vitro fertilization. *Fertil. Steril.* **95**(6), 1970–1974. 10.1016/j.fertnstert.2011.01.154 (2011). 21353671 10.1016/j.fertnstert.2011.01.154 PMC3080431

[41] Oviedo-Ojeda, M. F. et al. Effect of supplementation with different fatty acid profile to the dam in early gestation and to the offspring on the finishing diet on offspring growth and hypothalamus mRNA expression in sheep. *J. Anim. Sci.* **99**(4), skab064. 10.1093/jas/skab064 (2021). 33640974 10.1093/jas/skab064 PMC8075118

[42] Kujoana, T. C., Mabelebele, M. & Sebola, N. A. Role of dietary fats in reproductive, health, and nutritional benefits in farm animals: A review. *Open Agric.* **9**, 20220244. 10.1515/opag-2022-0244 (2024).

